# Craspedocephalus trigonocephalus Envenomation in the United States: A Case Report

**DOI:** 10.7759/cureus.88531

**Published:** 2025-07-22

**Authors:** Hayley T Gartner, Reeves E Simmons, Dawn R Sollee, Shahnaz Rashid, Sophia Sheikh

**Affiliations:** 1 Florida/United States Virgin Islands Poison Information Center Jacksonville, University of Florida Health, Jacksonville, USA; 2 Emergency Medicine, University of Florida College of Medicine-Jacksonville, Jacksonville, USA

**Keywords:** antivenom, envenomation, exotic animal, international, medical toxicology, poison control, snake

## Abstract

Exotic snake envenomations are increasingly encountered in the United States (US) due to the growing ownership of non-native venomous reptiles. We report the case of a 49-year-old man bitten by his own Sri Lankan green pit viper (*Craspedocephalus trigonocephalus, *formerly *Trimeresurus trigonocephalus*). The patient presented to the emergency department (ED) with rapid-onset, severe local pain and swelling extending from the bite site on his finger into the wrist. He also reported dizziness and numbness. Laboratory results were within normal limits. Poison control was contacted, and a clinical toxicologist was consulted early in the course. They accessed the Antivenom Index to identify a cross-reactive green pit viper antivenom, which was sourced from a local antivenom bank. Six vials of Hemato Polyvalent Snake Antivenin (Queen Saovabha Memorial Institute, Bangkok, Thailand) were administered intravenously (IV) five hours after envenomation. Swelling subsequently stabilized, and the patient remained hemodynamically stable with no progression of signs or symptoms. He was discharged in good condition after 26 hours of hospital observation. To our knowledge, there are currently no published case reports documenting the clinical use of Hemato Polyvalent Snake Antivenin (Queen Saovabha Memorial Institute) for this species. This case highlights the importance of early consultation with poison control and efficient coordination with institutional review boards (IRBs) and hospital pharmacies to secure life- and limb-saving treatment. Emergency clinicians should be aware of these protocols as exotic envenomations become more prevalent in the US.

## Introduction

Exotic snake envenomations are rare but increasingly reported across the United States (US) due to private ownership of non-native species [1]. Multiple cases involving foreign species have been documented from 2013 to 2022, with significant implications for emergency preparedness and treatment logistics [2]. The Sri Lankan green pit viper (*Craspedocephalus trigonocephalus*, formerly *Trimeresurus trigonocephalus*) is a venomous pit viper, with venom known to cause severe local tissue effects and hematologic disturbances [3,4]. Recommended antivenoms for treating envenomations from this species, including Hemato Polyvalent Snake Antivenin (Queen Saovabha Memorial Institute, Bangkok, Thailand) and Green Pit Viper Antivenom (Queen Saovabha Memorial Institute), are not approved for use within the US [5]. Their use requires an Investigational New Drug (IND) application, emergency institutional review board (IRB) approval, and coordination with regional antivenom banks or zoos, potentially leading to delayed treatment and adverse patient outcomes. We are unaware of published reports describing the management of *Craspedocephalus trigonocephalus* (*C. trigonocephalus*) envenomation with antivenom.

## Case presentation

**Figure 1:**
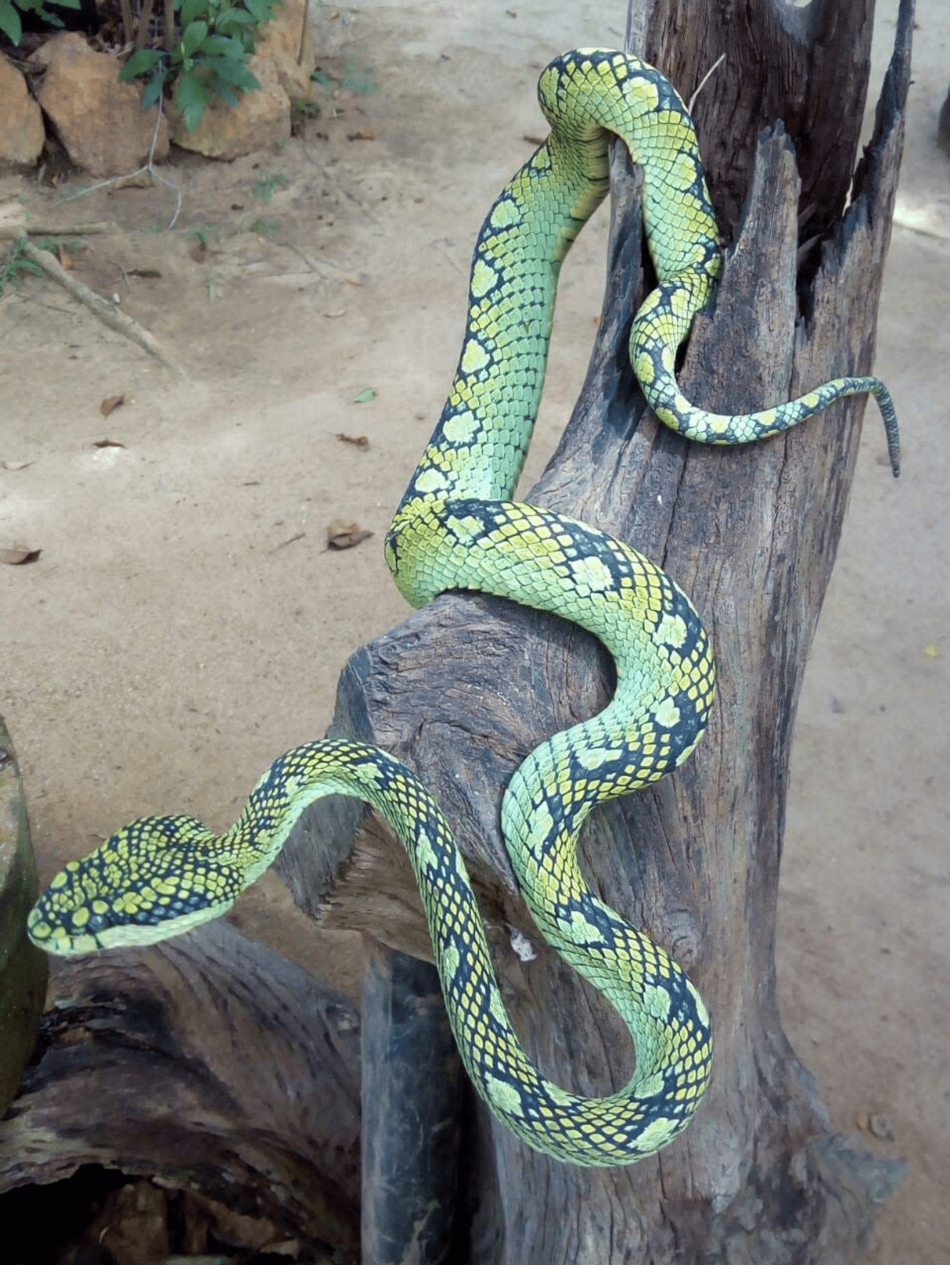
Craspedocephalus trigonocephalus (Sri Lankan green pit viper) Photo courtesy of Prof. Indika Gawarammana.

A 49-year-old man with a past medical history of hypertension on losartan presented to the emergency department (ED) 20 minutes following a bite to the tip of his left index finger by a *C. trigonocephalus *(Figure 1). He reported immediate severe pain, localized swelling, numbness, and dizziness. Vital signs on presentation were as follows: heart rate (HR) 78 beats per minute (bpm), blood pressure (BP) 173/102 millimeters of mercury (mmHg), respiratory rate (RR) 16 breaths per minute, temperature (temp) 98.3 degrees Fahrenheit (°F), and oxygen saturation (O₂ sat) 97% on room air. Initial laboratory tests were within normal limits: hemoglobin (Hgb) 15.5 grams per deciliter (g/dL), hematocrit (Hct) 46.3%, platelet count (Plt) 292 × 10³ per microliter (× 10³/μL), prothrombin time (PT) 12 seconds, international normalized ratio (INR) 0.9, partial thromboplastin time (PTT) 28.7 seconds, and fibrinogen 462 milligrams per deciliter (mg/dL) (Table 1). A radiograph of the bite site revealed no evidence of a foreign body. The patient's affected extremity was elevated above heart level, and tetanus prophylaxis was updated. Despite opioid analgesia, the patient’s pain remained severe. Over the next hour, swelling progressed from the finger into the wrist. Antivenom procurement was initiated in coordination with the regional poison control center and a local antivenom bank. Approximately five hours post-bite, six vials of Hemato Polyvalent Snake Antivenin (Queen Saovabha Memorial Institute) were administered off-label intravenously (IV), and no adverse effects were reported. Following antivenom administration, no further progression of swelling was observed, and the patient was admitted to the intensive care unit (ICU). Serial laboratory tests showed no development of coagulopathy. Upon discharge, laboratory parameters included: Plt 238 × 10³/μL, PT 12.7 seconds, INR 0.9, PTT 27.9 seconds, and fibrinogen 270 mg/dL (Table 1). The patient was monitored in the ICU and discharged on hospital day 2, approximately 26 hours post-bite, with return precautions. At the time of discharge, swelling had stabilized, and the patient reported marked improvement in pain. He was discharged in stable condition with no signs of progressive envenomation or systemic complications.

**Table 1:** Laboratory parameters

Parameter	Presentation Value	Discharge Value	Reference Range	Units
Hemoglobin	15.5	-	13.5–17.5 (male)	g/dL
Hematocrit	46.3	-	41–53 (male)	%
Platelets	292 × 10³	238 × 10³	150–400 × 10³	/μL
Prothrombin time	12.0	12.7	11.0–13.5	seconds
International normalized ratio	0.9	0.9	0.8–1.2	-
Partial thromboplastin time	28.7	27.9	25–35	seconds
Fibrinogen	462	270	200–400	mg/dL

## Discussion

Poison control centers, medical and clinical toxicologists, herpetologists, and toxinologists possess specialized expertise regarding the management of exotic snake envenomations, along with the understanding of antivenom composition, which is crucial when species-specific antivenom is unavailable [6,7]. Although Hemato Polyvalent Snake Antivenin (Queen Saovabha Memorial Institute) is specifically formulated for *Trimeresurus albolabris* envenomation (along with *Calloselasma rhodostoma* and *Daboia russelii siamensis*), it has demonstrated in vitro cross-neutralization against several congeneric heterologous venoms, including *C. trigonocephalus*, and is recommended by the World Health Organization as a potential treatment option [8-11]. However, its efficacy against the procoagulant effects of *C. trigonocephalus *venom appears to be the lowest among the activities assessed [3,8]. In a four-year prospective study of seventeen *C. trigonocephalus* bites, most occurred in estate workers during daytime and involved the upper limbs. Nearly all patients developed local symptoms, while systemic effects such as coagulopathy occurred in a minority [3]. To our knowledge, there are currently no published case reports documenting the clinical use of Hemato Polyvalent Snake Antivenin (Queen Saovabha Memorial Institute) for this species, potentially due to limitations in accessibility in Sri Lanka [3]. In the present case, given the patient’s signs and symptoms, the decision was made to administer the antivenom, which was readily available from a local antivenom bank. Antivenom banks are centralized repositories, typically maintained by medical or public health institutions, that store and distribute antivenoms for rapid access in the treatment of venomous bites and stings; poison control centers serve as a key resource for identifying and coordinating access to these banks within their regions.

This case underscores the critical importance of prompt coordination with poison control centers, rapid identification and procurement of appropriate antivenom, and strict adherence to regulatory protocols for the emergency use of foreign biologics. Although rare, exotic envenomations are becoming increasingly common in US EDs due to the rise in exotic pet ownership and interstate animal trade [1]. When Food and Drug Administration (FDA)-approved antivenom is not available or does not exist, the FDA permits the emergency use of unlicensed antivenoms through its IND application process [12]. The lending institution typically files the IND application upon importation, but the treating hospital must obtain IRB approval for emergency use and submit a case summary to the FDA’s Center for Biologics Evaluation and Research [13]. The FDA allows one-time emergency administration of foreign antivenom for life-threatening or seriously disabling conditions; subsequent doses require IRB approval, and the investigator must notify the IRB within five days [13]. This comprehensive response enabled expedited treatment and a positive outcome in this high-risk scenario.

## Conclusions

This case highlights the complex challenges associated with managing exotic snake envenomations in the US, particularly when species-specific antivenoms are unavailable. It reinforces the indispensable role of poison control centers and medical and clinical toxicologists in guiding evidence-based treatment decisions and navigating the regulatory landscape for emergency use of foreign biologics. Despite limited efficacy data, Hemato Polyvalent Snake Antivenin (Queen Saovabha Memorial Institute) was successfully used in this case due to its documented cross-reactivity and availability, illustrating the importance of clinical judgment in the absence of established protocols. As exotic envenomations become increasingly relevant, emergency physicians must be prepared to rapidly coordinate with specialized resources and adhere to FDA guidelines for investigational treatments. Ongoing documentation of such cases is essential to build a broader evidence base and inform future clinical management strategies.
